# Prediction of human health risk and disability-adjusted life years induced by heavy metals exposure through drinking water in Fars Province, Iran

**DOI:** 10.1038/s41598-023-46262-1

**Published:** 2023-11-04

**Authors:** Majid Radfard, Hassan Hashemi, Mohammad Ali Baghapour, Mohammad Reza Samaei, Masud Yunesian, Hamed Soleimani, Abooalfazl Azhdarpoor

**Affiliations:** 1 Department of Environmental Health Engineering, School of Public Health, Shiraz University of Medical Sciences, Shiraz, Iran; 2 Department of Environmental Health Engineering, School of Public Health, Tehran University of Medical Sciences, Tehran, Iran; 3 Student’s Scientific Research Center, Tehran University of Medical Sciences, Tehran, Iran

**Keywords:** Ecology, Environmental sciences

## Abstract

Exposure to heavy metals in contaminated drinking water is strongly correlated with various cancers, highlighting the burden of disease. This study aimed to assess the non-carcinogenic and carcinogenic risks associated with exposure to heavy metals (As, Pb, Cd, and Cr) in drinking water of Fars province and evaluate the attributed burden of disease. Non-carcinogenic risk assessment was performed using the hazard quotient (HQ) method, while the carcinogenic risk assessment utilized the excess lifetime cancer risk approach. The burden of disease was evaluated in terms of years of life lost, years lived with disability, and disability-adjusted life years (DALY) for three specific cancers: skin, lung, and kidney cancer. The average drinking water concentrations of arsenic (As), cadmium (Cd), chromium (Cr) and lead (Pb) were determined to be 0.72, 0.4, 1.10 and 0.72 μg/L, respectively. The total average HQ of heavy metals in drinking water in the study area were 0.127, 0.0047, 0.0009 and 0.0069, respectively. The average ILCRs of heavy metal in the entire country were in the following order: 1.15 × 10^−5^ for As, 2.22 × 10^−7^ for Cd and 3.41 × 10^−7^ for Cr. The results also indicated that among the various counties analyzed, Fasa experiences the greatest burden of disease in terms of DALYs, with a value of 87.56, specifically attributed to cancers caused by exposure to arsenic. Generally, it can be said that the burden of disease is a critical aspect of public health that requires comprehensive understanding and effective intervention.

## Introduction

Providing safe and adequate drinking water plays an important role in health promotion and reducing the environmental burden of disease. Universal and equitable access to safe and affordable drinking water for all by 2030 have been considered as the Sustainable Development Goal target 6.1. However, the presence of heavy metal contamination in drinking water sources has become a pressing global concern. Heavy metals, such as arsenic (As), cadmium (Cd), chromium (Cr), lead (Pb), can can enter water sources through natural geological processes or human activities such as mining, industrial discharge, and improper waste disposal,posing significant health risks to populations worldwide^1,2^.

Long-term exposure to heavy metals in drinking water, such as arsenic, cadmium, and chromium, has consistently been linked to various cancers like skin, lung, and kidney cancer^3^. These metals can enter water naturally or through human activities, gradually accumulating over time and posing significant health risks. Their buildup in the body leads to chronic toxicity, disrupting normal cellular functions, causing organ damage, weakening the immune system, and increasing susceptibility to diseases. Areas with contaminated drinking water face serious public health challenges, emphasizing the urgent need to reduce exposure to these well-known human carcinogens and protect overall population health^4,5^. The recommended drinking water standards set by regulatory bodies for heavy metals are as follows: 10 µg/L for As, 3 µg/L for Cd, 10 µg/L for Pb, and 50 µg/L for Cr^6–8^.

Prolonged exposure to specific heavy metals has been correlated with the onset of various cancers, including those affecting the skin, lungs, and kidneys. The gradual buildup of these metals within the body can lead to persistent toxic effects. Even minimal exposure levels can result in their gradual accumulation in tissues, disrupting normal cellular operations and heightening the likelihood of diseases, particularly cancers^9,10^. Extended contact with elevated levels of arsenic in drinking water is linked to escalated risks of cancers, cardiovascular ailments, neurodevelopmental disorders, and unfavorable reproductive outcomes. Likewise, exposure to cadmium has been associated with cancers affecting the lungs, prostate, kidneys, and breasts, with the World Health Organization (WHO) designating cadmium as a confirmed human carcinogen. Taking action to mitigate heavy metal contamination in drinking water sources is imperative for safeguarding public health from these detrimental repercussions. One widely adopted gauge for quantifying the impact of diseases and risk factors on overall population health is the disability-adjusted life years (DALY) metric^6,11,12^.

The burden of disease refers to the overall impact of a particular health condition on a population, encompassing not only mortality but also morbidity and the social and economic consequences of illness. The use of the disability-adjusted life years (DALY) metric in evaluating the risk and burden of disease caused by exposure to heavy metals through drinking water is of utmost significance in the field of public health. The DALY combines two components: years of life lost (YLL) and years lived with disability (YLD)^13^. YLL represents the number of years lost due to premature death, while YLD accounts for the years lived with a disability or in a less than optimal health state. By summing these components, DALY provides a comprehensive estimation of the overall impact of a particular disease or risk factor on a population^14,15^. By encompassing a wide range of health outcomes, including physical, psychological, and social dimensions of diseases, DALY offers a comprehensive measure of the societal impact resulting from heavy metal exposure.

This approach aids in prioritizing interventions and allocating resources effectively to address identified health risks^16,17^. To date, several studies have reported associations between long-term exposure to certain heavy metals and the development of cancers, cardiovascular diseases, neurodevelopmental disorders, and adverse reproductive outcomes. Nevertheless, there is a scarcity of studies that specifically address Iranian provinces, despite the distinctive environmental and socio-economic factors in the country that could potentially affect the presence of heavy metals in drinking water sources^18,19^. By conducting this evaluation in different parts of Iran, we can contribute valuable insights to the limited body of knowledge on heavy metal exposure and its health consequences in this context. Furthermore, while previous studies have recognized the importance of the DALY metric in assessing the burden of disease, few have applied it directly to heavy metal exposure through drinking water^20,21^.This approach allows us to compare the burden of disease caused by heavy metal exposure to other health risks, prioritize interventions, and evaluate the cost-effectiveness of preventive measures.

## Materials and methods

### Study area

**Figure 1:**
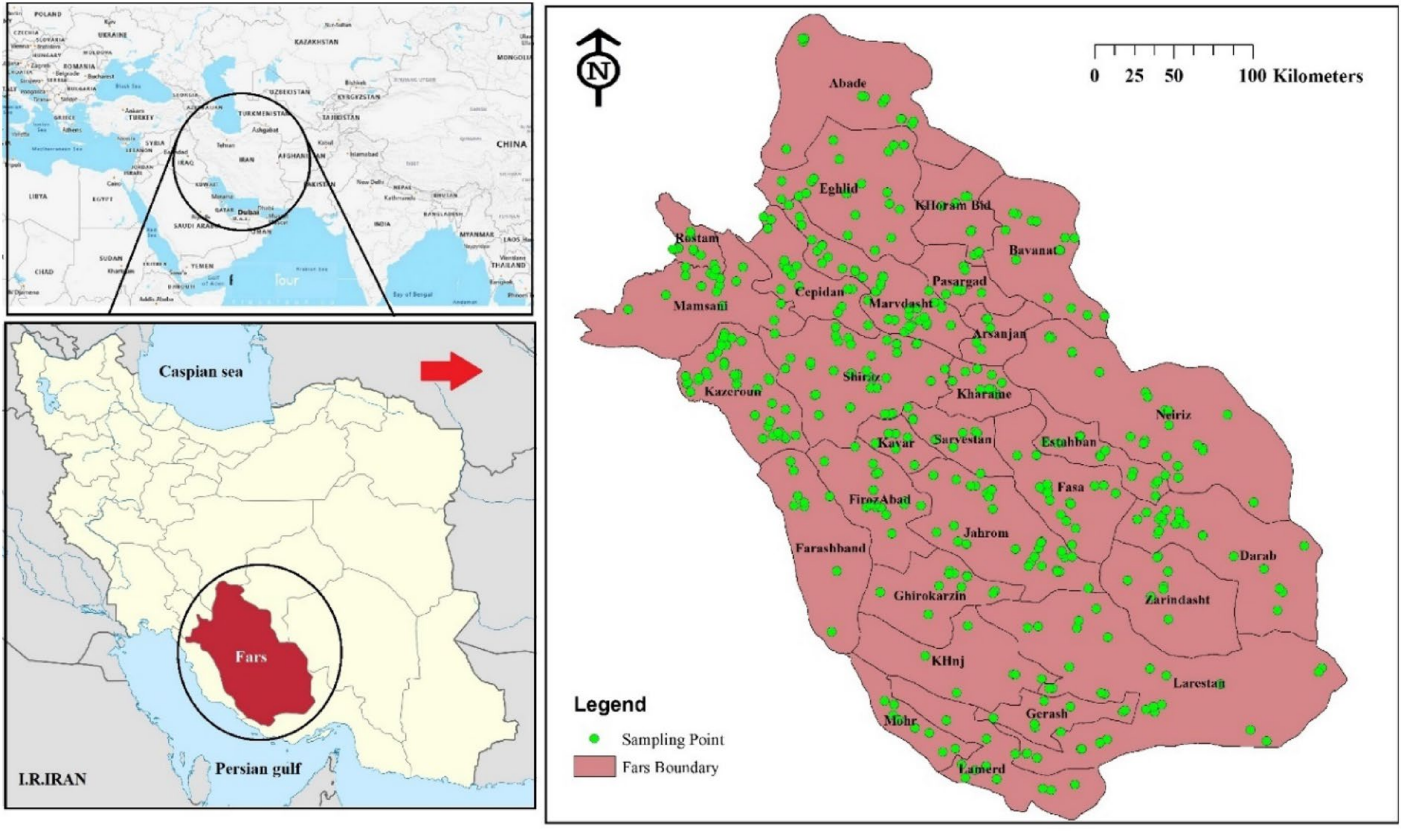
The location map of the studied area and sampling sites was generated using ArcGIS 10.4 software http://appsforms.esri.com/products/download/index.cfm?fuseaction=download.main&downloadid=1932.

Fars province, situated in the southwest of Iran, encompasses an area of 122,400 km^2^. It shares its borders with six adjacent provinces: Isfahan to the north, Kohgiluyeh and Boyer-Ahmad to the west, Bushehr to the south, Hormozgan to the southwest, and Yazd and Kerman to the east. The topography of Fars province is marked by diverse features, including mountain ranges, deserts, and fertile plains. Mount Dena, towering at an impressive height of 4409 m, stands as the highest peak in the region. Figure 1 presents the geographical map of the study area, highlighting the Fars province in southwest Iran.

The geological composition of Fars province comprises various formations, including Fars, Aghajari, Bakhtiari, Bangestan, and Sachun. These formations consist of marl, limestone, gypsum, andesite, sandstone, and limestone, respectively. This geological diversity plays a crucial role in the hydrogeological dynamics of the region. Additionally, the province features several significant rivers, including the Kor River and the Dez River.

With an approximate population of 4.9 million people, Fars province is primarily inhabited by Persian communities. The climate exhibits regional variations, with moderate conditions prevailing in the northern areas, while the southern regions experience hot and arid climates. The average annual rainfall stands at around 330 mm, with higher precipitation occurring in mountainous areas. It is noteworthy that the province experiences considerable temperature fluctuations due to climate variability. For instance, colder regions witness temperatures ranging from − 15 °C during the cold season to 26 °C during the hot season, while hotter and drier regions experience temperatures ranging from 4 °C to a scorching 48 °C during the respective seasons. These climatic factors contribute to the unique environmental characteristics of Fars province, which are crucial considerations in assessing water-related issues and their impact on public health. The groundwater level in the region has undergone a significant transition, decreasing from 1545 m in 2004 to less than 200 m in the year of conducting this study, 2021. This substantial alteration in groundwater levels underscores the dynamic nature of the hydrogeological system in Fars province, emphasizing its relevance in the context of the research conducted.

Furthermore, it is imperative to highlight that a substantial portion of the population in Fars province relies on well groundwater for their drinking water needs, accounting for approximately 79 percent of the available resources (Iran Water Resources Management Company, [Year]). This reliance underscores the critical importance of assessing heavy metal pollution in well groundwater sources, as it directly impacts a significant segment of the population. Given this context, our research plays a crucial role in evaluating the associated health risks and providing essential insights for public health interventions and policy decisions.

### Sampling and data collection

Water samples were collected from a total of 569 wells in the drinking water supply system of 28 cities located within Fars province (Fig. 1). At each sampling point, two separate water samples were collected using 2-L polyethylene containers. Prior to sampling, the water was allowed to flow for 2 min, and the sampling bottles were carefully filled. One bottle was filled without the addition of acid and bubbles, while the other bottle was rinsed with a solution of double-distilled water and nitric acid in a 1:1 ratio. The pH of the samples was adjusted to less than 2 using pure nitric acid (E. Merck, Darmstadt, Germany) to minimize the absorption of heavy metals in the container lining and stabilize microbial activity. Following sample collection, the water samples were transferred to the laboratory while maintaining a temperature of 4 °C.

The heavy metal concentration data for drinking water in both rural and urban communities were obtained from the 2020 drinking water quality database of the Center of Environmental and Occupational Health, Ministry of Health and Medical Education. This database is not publicly accessible. The selected heavy metals for this study were arsenic (As), cadmium (Cd), chromium (Cr), and lead (Pb) due to their severe health effects and potential presence in high concentrations in drinking water. The collection of drinking water samples for heavy metal measurements was conducted seasonally by environmental health officers throughout the country. The total number of drinking water samples collected for heavy metal measurements was approximately 569, with sample sizes in each community proportionate to the population. The heavy metal measurements were performed using the atomic absorption spectrophotometry (AAS) in water quality laboratories of the Ministry of Health and Medical Education, following the instructions outlined in the Standard Methods for the Examination of Water and Wastewater^2,22^.

To ensure data quality, the measurement data of heavy metal concentrations underwent a cleaning process based on the methods described by. Outliers were identified and removed from the dataset. Subsequently, the heavy metal concentration data were categorized by community, and the arithmetic mean and standard deviation of heavy metal concentrations in drinking water were calculated. These calculated values were utilized to assess the exposure dose, health risk, and attributable burden of disease associated with heavy metal contamination in the study area^23,24^.

### Chemical analysis

In the chemical analysis section, the collected water samples were subjected to rigorous laboratory testing to determine the concentrations of heavy metals and other relevant physical and chemical parameters. The analysis of heavy metals, including arsenic (As), cadmium (Cd), chromium (Cr), and lead (Pb), was conducted using established methods (atomic absorption spectrophotometry (AAS)) in accordance with the Standard Methods for the Examination of Water and Wastewater^22^. These methods provide accurate and reliable measurements of heavy metal concentrations in water samples. To ensure the accuracy and reliability of the analysis, several quality control measures were implemented. Standard samples and controls were analyzed after every 10 samples to assess the reliability and repeatability of the analysis. These measures help in identifying any potential variations or inconsistencies in the results, thereby ensuring the overall quality of the data. The analysis of the contaminant contents in the water samples was conducted using graphite furnace atomic absorption spectrometry (Perkin Elmer AA-Analyst 200), which is a reliable method for precise measurements of the concentration levels of contaminants. It is important to note that the utilization of double-distilled water and the preparation of standard solutions contribute to the accuracy and reliability of the analysis. These steps minimize any potential interference or contamination during the analysis process, ensuring that the obtained results are representative of the actual concentrations of the contaminants in the water samples^8,25^.

### Risk assessment

Risk management involves assessing the probability of an incident occurrence and the potential adverse health effects on humans and other animals exposed to environmental risk factors. Risk assessmnet was calculated using the modified Eqs. (1) and (2) provided below to estimate the average daily dose (ADD) of carcinogenic and non-carcinogenic elements:^2,4^.

1 $$ {\text{ADDc}} = \frac{{{\text{C}} \times \left( {{\text{RBA}} \times {\text{IR}}} \right) \times {\text{EF}} \times {\text{EDc}}}}{{{\text{BW}} \times {\text{ATc}}}} $$

2 $$ {\text{ADDnc}} = \frac{{{\text{C}} \times \left( {{\text{RBA}} \times {\text{IR}}} \right) \times {\text{EF}} \times {\text{EDcn}}}}{{{\text{BW}} \times {\text{ATnc}}}} $$

where ADDc: The average daily dose (carcinogenic elements (mg/kg/day)), ADDnc: The average daily dose (non-carcinogenic elements (mg/kg/day)), C: The contamination concentration (mg/L), RBA: Relative biological availability, IR: The ingestion rate (L/day), EDc: The exposure duration (carsinogenic), Ednc: The exposure duration (non-carcinogenic), BW: The body weight (kg), ATc: The average time for cancer risk assessment (day), ATnc: The average time for non-cancer risk assessment (day).

Table 1 provides an overview of the exposure parameters considered in the risk assessment calculations.

**Table 1:** Input parameters to characterize the ADD, ELCR, HQ value^2,4,26^.

Exposure parameters	Symbols	Units	Value
Concentration of water	C	µg/l	Table 6
Exposure frequency	EF	Days/year	365
Relative biological availability	RBA	–	Table 2
Relative source contribution	RSC	–	Table 2
Exposure duration	ED	Years	–^a^
Average time cancer	ATc	Days	–^b^
Average time non-cancer	ATnc	Days	–^c^
Body weight	BW	Kg	–^d^
Ingestion rate	IR	L/day	–^e^
Reference dose	RFD	(Mg/Kg/Day)	Table 2
Cancer slope factor	CFS	(Mg/Kg/Day)	Table 2

^a^Exposure duration for adults 70 years and for children 10 years.

^b^Average Time cancer for adults exposure duration cancer * exposure frequency.

^c^Average time cancer for adults exposure duration cancer * exposure frequency.

^d^Body weight for adults 72 kg and for children 32.7 kg.

^e^Ingestion rate for adults 2 l and for children 1 l.

In developing countries, especially those with warmer climates, the amount of water used as a criterion to calculate exposure differs significantly from the values of water indicators used by the World Health Organization (WHO) to determine the guideline values for drinking water pollutants. Hence, this study leveraged water usage data from Khan's study, which pertains to a geographically proximate area, and integrated local demographic information such as age and weight. This approach was adopted to mitigate potential inaccuracies. The Exposure Frequency (EF) was determined based on recommendations from the United States Environmental Protection Agency (USEPA). The life expectancy at birth was considered as 70 years, corresponding to an average life of 2550 days^27,28^.

#### Non-carcinogenic risk assessment

Non-carcinogenic risk assessment is an essential component of this study, aiming to evaluate the potential health risks posed by the metals present in drinking water. It is as assessed using a non-carcinogenic risk factor known as the Hazard Quotient (HQ), which is calculated as follows:

3 $$ {\text{HQ}}\left( {{\text{Hazard}}\;{\text{quotient}}} \right) = \frac{{{\text{ADDnc}}}}{{{\text{RFD}} \times {\text{RSC}}}} $$

ADDnc: The average daily dose of metal in drinking water for non-carcinogenic elements (mg/kg/day), RFD: Reference dose (µg/kg/day), RSC: Relative source contribution.

The reference for metal’s doses, As, Cr, Pb, and Cd are presented (Tables 1, 2). If the HQ is more than 1 there is the possibility of non-carcinogenic effects on health, while if HQ is less than or equal to 1 is likely residents will not be considered of any health risks resulting of exposure to the elements^29–31^.

**Table 2:** The toxicity responses to heavy metals and metalloid as the oral reference dose (RfD) and oral slope factor (SF)^2,26,36–38^.

Heavy metals/metalloid	Oral RFD (Mg/Kg/Day)	Oral CSF (Mg/Kg/Day)	RBA	RSC
Cd	5.00E−04	0.38	0.05	0.25
Pb	3.50E−03	N.A	0.2	0.2
As	3.00E−04	1.5	0.37	0.2
Cr	3.00E−03	0.19	0.06	0.7

*N.A* Not available.

#### Carcinogenic risk assessment

Carcinogenic and non-carcinogenic risk assessments are essential components of this study, focusing on the evaluation of potential health risks associated with chromium (Cr), cadmium (Cd), arsenic (As), as well as lead (Pb) as a non-carcinogenic element. The classification of these elements as carcinogens and the non-carcinogenic risks are based on the guidelines provided by the United States Environmental Protection Agency (EPA) and the International Agency for Research on Cancer (IARC)^26,32,33^.To assess the carcinogenic risk, parameters such as the oral reference dose (RfD) and oral slope factor (CSF) are considered for chromium, cadmium, and arsenic, as presented in Table 2. These values provide insights into the potential cancer risks associated with exposure to these elements^4,34,35^.

The carcinogenic potential of these elements is determined by calcualating the excess lifetime cancer risk (ELCR) using the following formula:

4 $$ {\text{ELCR}} = {\text{ADD}}_{{\text{C}}} \times {\text{CSF}} $$

where ELCR: Excess lifetime cancer risk, ADDc: Average daily doses (mg/kg/day), CSF: Cancer slope factor) mg/kg/day).

By calculating the ELCR, we can estimate the excess risk of developing cancer over a lifetime due to exposure to the identified carcinogenic elements. The calculated ELCR will be compared to the acceptable maximum risk suggested by the USEPA, which is ≤ 1 × 10–6. If the calculated ELCR exceeds this threshold, it indicates a potential health risk to the exposed residents. Additionally, the non-carcinogenic risk assessment includes lead (Pb), which is not considered a carcinogen through the ingestion pathway of drinking water. The potential non-carcinogenic risks associated with lead exposure will be evaluated using the hazard quotient (HQ) approach, calculated as described earlier (Eq. 3)^39–41^.

### Burdn of desease attributable to heavy metals

The burden of disease associated with the intake of heavy metals through drinking water was assessed in terms of years of life lost due to premature mortality (YLL), years lived with disability (YLD), and disability-adjusted life years (DALY)^42^. For precise estimation of the disease burden linked to heavy metal exposure, a two-stage disease model was deployed. This model encompasses a treatment phase and a subsequent mortality phase for the associated cancers. This model provided a more comprehensive understanding of the impact of heavy metal exposure on the development and outcomes of specific cancers.The treatment phase of the disease model consisted of two distinct stages: 1. Diagnosis and treatment, and 2. remission to cure.

During the diagnosis and treatment stage, individuals undergo medical examinations, receive appropriate treatments such as surgery, chemotherapy, or radiation therapy, and work towards suppressing the cancerous growth. After undergoing successful treatment, individuals progress into the remission-to-cure stage, characterized by a period of recuperation and vigilant monitoring to verify the absence of cancer recurrence^43–45^. The death phase of the disease model incorporated multiple stages that reflect the progressive nature of cancer and its ultimate outcome^46^. It encompassed: 1. The diagnosis and treatment stage, similar to the treatment phase, as individuals may continue to receive medical interventions in an effort to manage the disease, 2. The remission to death stage acknowledged the unfortunate scenario where cancer reemerges despite prior remission efforts, leading to a deterioration of health and eventual mortality^47,48^, 3. The pre-final phase showed the advanced stage of the disease, where individuals may experience severe symptoms and complications, necessitating palliative care and supportive treatments, Finally, 4. the final phase denoted the terminal stage of the disease, reflecting the end-of-life period characterized by significant decline in overall health^16,49,50^.

By incorporating these distinct phases into the disease model, a more detailed and realistic assessment of the burden of disease resulting from heavy metal exposure was achieved. This approach offered comprehensive insights into the complete trajectory of cancer, encompassing initial diagnosis, treatment, potential remission, and the subsequent progression ultimately culminating in mortality. This approach allowed for a comprehensive evaluation of the impact of heavy metal exposure on different stages of cancer and facilitated a more accurate estimation of the associated burden of disease.The YLL, YLD, and DALY parameters were calculated by the following equations^14^:

5 $$ {\text{DALY}}_{{{\text{i}}.{\text{s}}.{\text{r}}}} = {\text{YLL}}_{{{\text{i}}.{\text{s}}.{\text{r}}}} + {\text{YLD}}_{{{\text{i}}.{\text{s}}.{\text{r}}}} $$

6 $$ {\text{YLL}}_{{{\text{i}}.{\text{s}}.{\text{r}}}} = \frac{{{\text{ILCR}}_{{{\text{i}}.{\text{s}}.{\text{r}}}} }}{70} \times {\text{P}}_{{{\text{s}}.{\text{r}}}} \times \left( {1 - {\text{SR}}_{{\text{c}}} } \right) \times \left( {{\text{L}} - \left( {{\text{a}}_{{{\text{s}}.{\text{r}}}} + {\text{D}}_{{{\text{c}}.{\text{dt}}}} + {\text{D}}_{{{\text{c}}.{\text{rd}}}} + {\text{D}}_{{{\text{c}}.{\text{pt}}}} + {\text{D}}_{{{\text{c}}.{\text{t}}}} } \right)} \right) $$

7 $$ \begin{aligned} YLD_{i.s.r} & = \frac{{ILCR_{i.s.r} }}{70} \times P_{s.r} \times \left( {DW_{c.dt} \times D_{C.dt} + SR_{c} \times DW_{C.rc} \times D_{c.rc} + \left( {1 - SR_{c} } \right)} \right. \\ & \quad \left. { \times \;DW_{c.rd} \times D_{c.rd} + \left( {1 - SR_{c} } \right) \times DW_{c.pt} \times D_{c.pt} + \left( {1 - SR_{c} } \right) \times DW_{c.t} \times D_{c.t} } \right) \\ \end{aligned} $$

where DALYi,s,r (y): The disability-adjusted life years induced by exposure to heavy metal i through drinking water for sex s in region r, YLLi,s,r (y): The years of life lost due to premature mortality induced by exposure to heavy metal i through drinking water for sex s in region r, YLDi,s,r (y): The years lived with disability induced by exposure to heavy metal i through drinking water for sex s in region r, Ps,r (person): Population of the study area, SRc (dimensionless): The survival rate of cancer c, L (y): Life expectancy in the study area (74.2 y), As,r: Average age in the population of the study area (32.5 y), Dc,dt, Dc,rc, Dc,rd, Dc,pt, and Dc,t: Respectively; the duration of diagnosis and treatment phase of cancer c, duration of remission to cure phase of cancer c, duration of remission to death phase of cancer c, duration of the pre-final phase of cancer c, and duration of the final phase of cancer c, DWc,dt, DWc,rc, DWc,rd, DWc,pt, and DWc,t: Respectively; the disability weight of diag- nosis and treatment phase of cancer c, disability weight of remission to cure phase of cancer c, disability weight of remission to death phase of cancer c, disability weight of pre-final phase of cancer c, and disability weight of final phase of cancer c.

In summary, YLL focuses on premature mortality, capturing the years of life lost due to early death caused by heavy metal-related cancers. Years of Life Lost (YLL) are computed based on the population that is at risk or affected by a specific health condition or cause of death. On the other hand, YLD focuses on the impact of disability caused by heavy metal-related cancers, capturing the years individuals live with compromised health and functioning. These measures collectively help assess the burden of disease associated with heavy metal exposure and inform public health interventions and policies^14,51,52^.

### Statistical analysis

All calculations such as average, standard deviation and ranges for the target parameters was done by using the Excel 2010 software. Statistical analysis such as correlation analysis was done by SPSS.V.11.5 software. Map of the study area was made using ARC GIS.V 10.4.

## Results and discussion

### Concentration of heavy metals

The results exhibit variations in mean and range values across different counties, indicating spatial differences in heavy metal contamination. The presence of heavy metals in drinking water can originate from various sources. Industrial activities, including mining, metal production, and waste disposal, are common culprits. The concentrations of heavy metals, including Arsenic (As), Lead (Pb), Chromium (Cr), and Cadmium (Cd) is presented in Table 3.

**Table 3:** Heavy metal concentration in different studied area.

County	AS (µg/l)	Pb (µg/l)	Cr (µg/l)	Cd (µg/l)
	Mean (range)	Mean (range)	Mean (range)	Mean (range)
Arsanjan	1.10 (0.94–1.27)	0.52 (0.38–0.67)	1.50 (1.4–1.6)	0.24 (0.21–0.26)
Estheban	0.56 (0.26–1.31)	0.68 (0.23–1.31)	1.36 (0.4–1.51)	0.38 (0.10–0.41)
Euclid	0.65 (0.27–1.15)	0.62 (0.31–1.17)	0.86 (0.61–1.27)	0.29 (0.12–0.41)
Abadah	0.29 (0.25–0.36)	0.67 (0.26–1.16)	1.15 (0.67–1.43)	0.17 (0.01–0.40)
Bowanat	0.94 (0.31–1.36)	0.58 (0.22–1.03)	1.05 (0.6–1.35)	0.25 (0.11–0.43)
Pasargad	0.26 (0.25–0.28)	0.30 (0.31–0.41)	0.85 (0.81–0.95)	0.90 (0.15–1.51)
Jahram	1.15 (0.26–2.5)	0.66 (0.21–2.1)	1.21 (0.66–3.59)	0.37 (0.14–1.43)
Kharameh	0.42 (0.26–0.86)	1.02 (0.27–1.9)	1.16 (0.91–1.43)	0.15 (0.10–0.21)
Khoram bid	1.26 (0.91–1.11)	0.73 (0.41–1.4)	1.41 (0.63–3.21)	0.33 (0.10–0.6)
khanj	0.36 (0.22–0.36)	0.31 (0.22–0.4)	1.19 (0.91–1.21)	0.53 (0.20- 0.53)
Darab	0.61 (0.27–1.2)	0.41 (0.21–0.9)	1.41 (0.69–2.26)	0.3 (0.21–0.75)
Rostam	0.41 (0.25–1.08)	0.86 (0.31–0.9)	1.35 (0.84–2.18)	0.31(0.15–1.1)
Zarin Dasht	0.71 (0.26–1.17)	0.76 (0.71–0.9)	0.66 (0.63–0.82)	0.21 (0.15–0.31)
Sepidan	0.71 (0.27–1.11)	0.73 (0.3–1.18)	0.84 (0.61–1.13)	0.7 (0.12–1.54)
Sarvestan	1.11 (0.27–2.4)	0.64 (0.27–1.38)	0.92 (0.39–1.35)	0.57 (0.18–1.47)
Shiraz	0.93 (0.26–3.2)	0.92 (0.23–3.75)	1.16 (0.66–3.54)	0.45 (0.12–1.2)
Farashband	0.90 (0.81–1.03)	0.56 (0.23–0.9)	1.60 (1.05–2.26)	0.21 (0.19–0.3)
Fasa	1.42 (0.85–2.37)	0.53(0.21–1.11)	1.26 (0.69–2.18)	0.22 (0.15–0.31)
Firozabad	0.50 (0.27–1.22)	0.60 (0.22–1.12)	0.79 (0.63–1.05)	0.31 (0.20–1.13)
Gairocazine	0.65 (0.26–1.17)	0.83 (0.65–1.26)	0.89 (0.71–0.98)	0.37 (0.1–0.82)
Kazeron	0.68 (0.27–2.7)	0.63 (0.21–1.4)	1.13 (0.39–3.54)	0.44 (0.12–1.58)
Quar	0.79 (0.26–1.31)	0.66 (0.41–0.91)	0.94 (0.75–1.11)	0.70 (0.24–1.17)
Grash	0.61 (0.28–0.94)	2.59 (1.38–3.75)	1.03 (0.61–1.43)	0.74 (0.15–1.34)
Larestan	0.934 (0.26–2)	0.64 (0.3–1.89)	1.32 (0.67–2.26)	0.56 (0.03–1.53)
Lamard	0.95 (0.91–1.1)	0.93 (0.9–1.2)	1.58 (0.95–2.18)	0.29 (0.2–0.4)
Marvdasht	0.47 (0.19–1.32)	0.62 (0.14–1.17)	0.91 (0.61–1.35)	0.66 (0.01–1.7)
Mamasani	0.54 (0.27–1.02)	0.88 (0.24–1.99)	0.77 (0.62–0.91)	0.40 (0.32–0.56)
Neyriz	0.63 (0.25–1.36)	0.67 (0.21–1.16)	1.24 (0.68–3.54)	0.49 (0.14–1.61)

**Figure 2:**
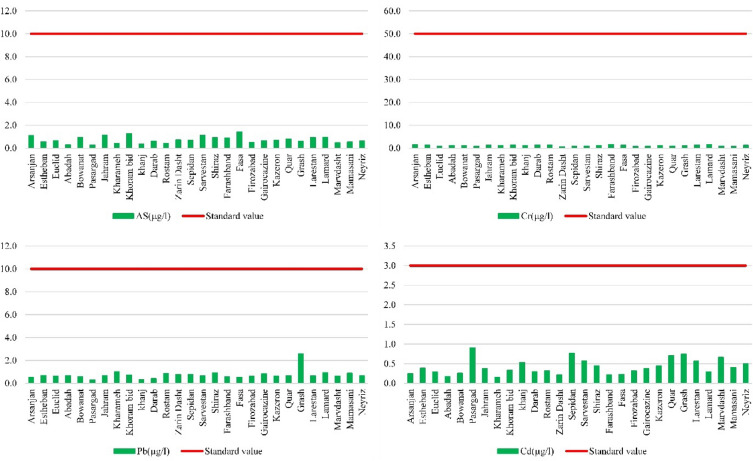
Average concentrations of heavy metals and comparing with WHO standard (red line).

The World Health Organization (WHO) has established maximum acceptable limits for As, Pb, Cr, and Cd in drinking water, set at 10, 50, 10, and 3 µg/L, respectively. According to the data in Table 3, the concentrations of As, Pb, Cr, and Cd in the studied counties were generally within acceptable ranges based on the WHO. In terms of As concentration, the mean values ranged from 0.26 (Pasrgad) to 1.42 (Fasa) across the different counties. Although no values exceeded the WHO guideline of 10 µg/L, some counties, such as Jahrom, Fasa, and Sarvestan exhibited relatively higher concentrations compared to other areas. However, it is important to consider that some counties exhibit slightly higher concentrations. The concentrations ranged from 0.14 to 3.75 µg/L for Pb, 0.39 to 3.54 µg/L for Cr, and 0.01 to 1.7 µg/L for Cd. While no values exceeded the WHO standard, it is crucial to remain vigilant and implement measures to prevent any future increase in heavy metal concentrations. The graph presented in Fig. 2 illustrates a comparison between the concentrations of As, Pb, Cr, and Cd in the studied counties and the corresponding WHO standards. This graph provides a visual representation of the extent to which the heavy metal concentrations comply with the recommended limits.

The origins of heavy metal contamination in the study area necessitate additional scrutiny. Potential sources may include industrial operations such as mining, metal production, and waste disposal, as well as agricultural practices involving the use of fertilizers containing heavy metals or the use of contaminated irrigation water. Furthermore, historical land use patterns could also serve as contributing factors to heavy metal contamination. Natural geological processes, such as leaching from rocks and soils, can also introduce heavy metals into water sources. Identifying these sources will aid in developing effective pollution prevention and control strategies to safeguard the health of the local population.the relatively narrow range of heavy metal concentrations observed in our study area can be attributed to several factors, including similar geological and environmental conditions, potential pollution sources, spatial proximity to neighboring regions, and the sampling design employed. These findings highlight the need for further investigation into the local factors influencing heavy metal contamination and provide valuable insights into the current status of heavy metal pollution in our study area.The study by Radfard et.al and also Mirzabeygi et.al et al. examined heavy metal contamination in nearby regions and reported comparable concentrations for Arsenic, Lead, Chromium, and Cadmium. These findings align with our results and provide additional evidence of the current status of heavy metal contamination in our study area^2,4^.

#### Saptial distribution of heavy metals

**Figure 3:**
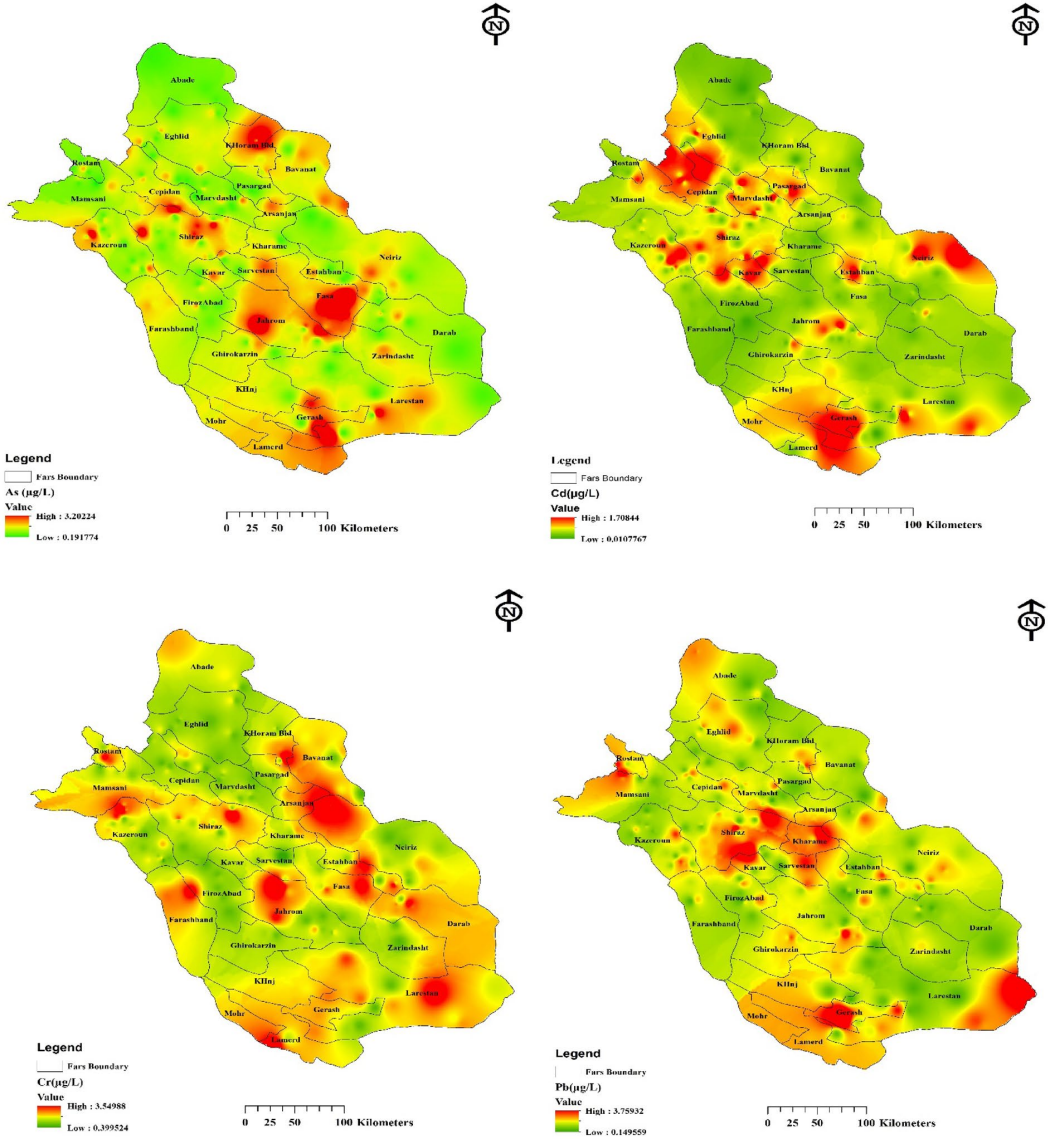
The spatial distribution of studied heavy metals was generated using ArcGIS 10.4 software http://orms.esri.com/products/download/index.cfm?fuseaction=download.main&downloadid=1932.

To facilitate a comprehensive understanding of the spatial distribution and visualize the concentrations, Geographic Information System (GIS) technology was employed. GIS offers a powerful tool for analyzing and presenting spatial data, allowing us to map the concentrations of heavy metals across different counties or regions. These zoning maps can assist in identifying areas that require closer monitoring and potential remediation efforts. According to Fig. 3, the measured concentrations of As range from 0.19 to 3.2 µg/L, while Cr concentrations range from 0.39 µg/L in Shiraz to 3.54 µg/L. The results also indicated that the concentrations of Cd vary between 0.01to 1.7 µg/L, with the highest levels observed in Marvdasht. Similarly, Pb concentrations range from 0.14 µg/l to 3.7 µg/l, with the highest levels found in Grash.These values reflect the varying levels of heavy metal contamination across different regions.

While the concentrations of all analyzed heavy metals in the study area are within acceptable limits, it is important to remain vigilant about the adverse health effects that these heavy metals can pose even at low levels of exposure. Totaly, our study provides comprehensive information on the concentrations of heavy metals in the study area, as well as their spatial distribution depicted through the GIS zoning map. While the recorded concentrations fall below the WHO guidelines, it is essential to recognize the potential health risks associated with heavy metal exposure in future investigations.

### Heavy metal health risk assessment

#### Non-carsinogenic risk assessment

**Figure 4:**
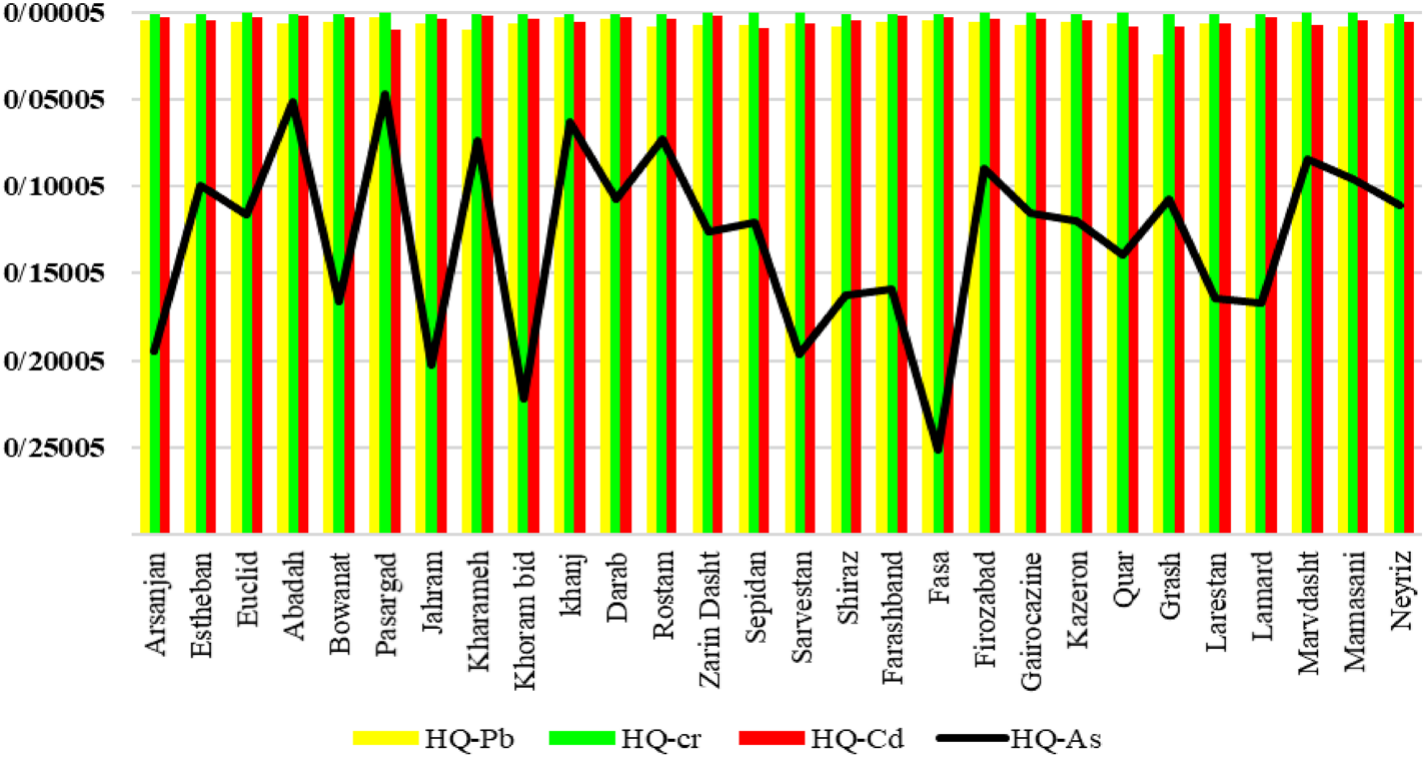
Population distribution of the HQs of exposure to heavy metals through drinking water by location.

The non-carcinogenic health risk assessment for heavy metals in drinking water was conducted using the Average Daily Dose (ADD) parameter, which was calculated using the modified Eq. 2. The equation takes into account factors such as contamination concentration (C), Relative Biological Availability (RBA), ingestion rate (IR), exposure duration (ED), body weight (BW), and average time for non-cancer risk assessment (ATnc). Figure 4 presents the results of HQ values of heavy metals (As, Pb, Cr, and Cd) in different counties.

In non-carcinogenic risk assessment of heavy metals, the hazard quotient (HQ) is a fundamental parameter used to evaluate the potential health risks associated with exposure to these substances. The Hazard Quotient (HQ) was calculated using the ADDnc (average daily dose of metal in drinking water for non-carcinogenic elements), Reference Dose (RFD), and Relative Source Contribution (RSC)^7^. HQ represents the ratio between the estimated exposure dose of a chemical and a reference dose (RfD) or a safe exposure limit established by regulatory agencies. The reference dose (RfD) is derived from toxicological studies and represents an estimate of the daily exposure level to a chemical that is unlikely to cause adverse health effects over a lifetime of exposure. It is usually based on the No Observed Adverse Effect Level (NOAEL) or the Lowest Observed Adverse Effect Level (LOAEL) determined from animal or human studies. The RfD takes into account factors such as uncertainty and variability in the data to ensure a conservative and protective estimate of safe exposure^53^.

It is crucial to highlight that the Hazard Quotient (HQ) methodology is frequently employed for non-carcinogenic risk assessment, as it specifically addresses potential adverse health effects apart from cancer. Carcinogenic risks linked with heavy metals are typically evaluated through distinct approaches, such as the utilization of cancer slope factors. If the resulting HQ value is greater than 1, it indicates a potential health risk, suggesting that the exposure dose of the chemical may exceed the safe limit set by the RfD^54^. HQ values were calculated for arsenic (HQ-As), lead (HQ-Pb), chromium (HQ-Cr), and cadmium (HQ-Cd). Arsenic levels, as measured by the HQ-As parameter, ranged from 0.046 to 0.251, which are relatively low.

The highest HQ-As value was observed in the county of Fasa (0.2513), indicating a relatively higher potential health risk associated with arsenic exposure in this area. On the other hand, the lowest HQ-As value was found in the county of Pasargad (0.0467), suggesting a comparatively lower risk of arsenic contamination^7,55^.

Exposure to elevated levels of arsenic can lead to adverse health effects such as increased cancer risk (skin, lung, bladder, kidney), cardiovascular diseases, respiratory problems, skin lesions, developmental issues in children, impacts on the nervous system, and negative effects on the liver, kidneys, and immune system^56^. Adhering to acceptable limits and minimizing exposure to arsenic are important for reducing these risks. Based on the our results, it could be observed that the HQ values are low and the concentration of arsenic is below the acceptable limits set by the WHO standard^7^. So the potential adverse health effects associated with arsenic exposure are generally considered to be minimal. The low HQ values indicate that the exposure to arsenic is unlikely to cause significant adverse health effects. However, it is essential to consider that long-term exposure to any level of arsenic may still have cumulative effects over time^57^.

Lead levels, as indicated by the HQ-Pb parameter, ranged from 0.002 to 0.024. The county of Grash exhibited the highest HQ-Pb valueaand the Pasargad showed the lowest HQ-Pb value, indicating a relatively lower risk of lead contamination. HQ-Cr parameter, ranged from 5.11837E−04 to 1.24469E−03. The county of Farashband demonstrated the highest HQ-Cr value, indicating a potential source of chromium pollution compare to other regions. HQ levels of Cd ranged from 0.001 (Kharameh) to 0.01 (Pasargad). Lead exposure can result in various adverse health effects, including neurological damage, developmental issues, cognitive impairment, cardiovascular problems, kidney damage, reproductive issues, and increased blood pressure.

High levels of chromium can lead to lung, nasal, and sinus cancer, respiratory problems, skin irritation, as well as liver and kidney damage. Cadmium exposure is associated with kidney damage, respiratory issues, weakened bone health, increased risk of lung cancer, and impacts on the cardiovascular and reproductive systems. The results of our study indicate that the concentrations of arsenic, lead, chromium, and cadmium in the studied area are within the acceptable limits set by the World Health Organization (WHO). Scientific databases and previous research also support our findings, showing that exposure to these heavy metals at the observed levels is unlikely to cause significant adverse health effects. However, it is essential to acknowledge that the potential health risks associated with heavy metal exposure can vary depending on factors such as individual susceptibility, duration of exposure, and cumulative effects over time. Therefore, continuous monitoring and regular assessments of heavy metal levels in the environment and human populations are necessary to ensure the long-term health and well-being of the community. Additionally, further research and epidemiological studies are warranted to explore any potential subtle or long-term health effects that may arise from chronic exposure to low levels of these heavy metals.

#### Carcinogenic risk assessment

**Figure 5:**
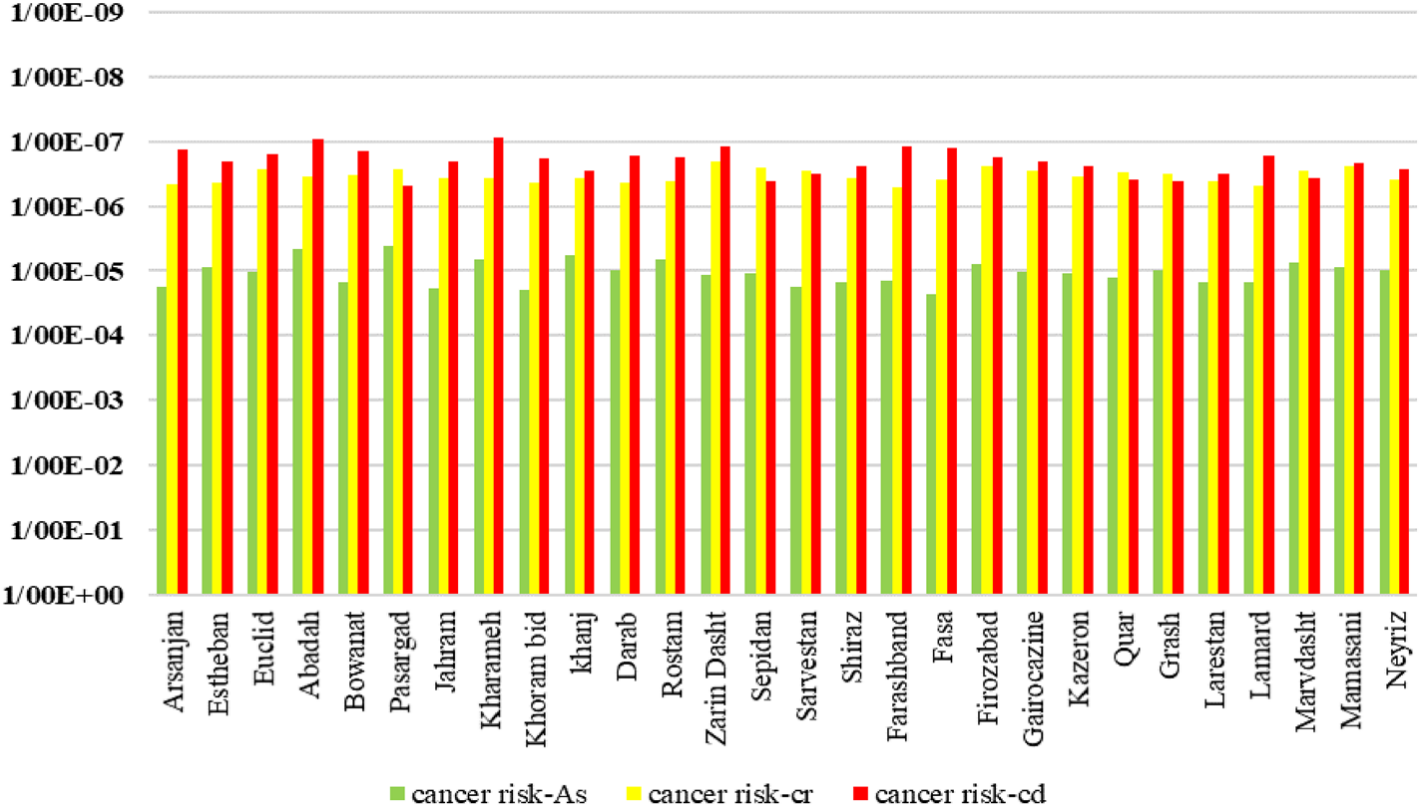
Average ELCRs of exposure to the heavy metals through drinking water by location.

In the field of toxicology, the carcinogenic risk assessment of heavy metals is often quantified using the Excess Lifetime Cancer Risk (ELCR) approach. The ELCR is a measure of the additional risk of developing cancer over a lifetime due to exposure to a particular carcinogenic substance, in this case, heavy metals. HQ and ELCR are both important metrics used in risk assessment, but they serve different purposes and assess different health outcomes. Indeed, the Hazard Quotient (HQ) primarily concentrates on non-cancer health effects, gauging the estimated exposure against a predetermined safe threshold level. Conversely, the Excess Lifetime Cancer Risk (ELCR) is specifically designed to assess the supplementary lifetime risk of developing cancer due to exposure to a carcinogenic substance^58^. The ELCR is calculated using the Average Daily Dose (ADD) and the Cancer Slope Factor (CSF). The Cancer Slope Factor (CSF) is a value determined through toxicological studies and represents the potency of a specific carcinogenic substance. It quantifies the increased cancer risk associated with a unit increase in the average daily dose of the heavy metal. Figure 5 presents ELCRs values of heavy metals (AS, Pb, Cr, and Cd) in different counties.

Generally, ELCR values below 1 in a million (10^−6^) are considered low risk, indicating a relatively low likelihood of developing cancer. ELCR values between 1 in a million and 1 in 10,000 (10^−6^ to 10^−4^) are considered moderate risk, while values above 1 in 10,000 (10^−4^) are considered high risk. As seen in Fig. 5**,** findings revealed significant variations in the carcinogenic risk levels across the studied counties. Arsenic, a highly toxic heavy metal in this study, exhibited varying levels of cancer risk. Among the three heavy metals, arsenic demonstrates the highest mean ELCR value of 1.672 × 10^−5^. Arsenic is a well-known carcinogen and is linked to various types of cancer, including skin, lung, bladder, and liver cancer. The elevated mean ELCR value for arsenic underscores the urgent need for effective mitigation strategies and stricter regulations to reduce exposure and protect the population from the associated cancer risks^7^.

The chromium exhibits a lower mean ELCR value of 3.494 × 10^−7^. This suggests a relatively lower cancer risk associated with chromium exposure compared to arsenic. Nonetheless, it remains crucial to acknowledge that chromium exposure continues to be a substantial concern, given its established association with an elevated risk of lung cancer. Mitigation measures and proper monitoring should be implemented to minimize exposure and reduce the potential cancer risks associated with chromium.

Cadmium, on the other hand, shows the lowest mean ELCR value among the studied heavy metals, with a value of 2.272 × 10^−7^. This indicates a relatively lower cancer risk associated with cadmium exposure compared to others. However, it is crucial to remain vigilant as cadmium exposure has been linked to lung and prostate cancer. Effective management practices, such as reducing cadmium emissions, implementing safety measures in industrial settings, and promoting awareness, should be prioritized to further minimize the cancer risks associated with cadmium.

Overall, the results of the carcinogenic risk assessment highlight the spatial variability in the potential health risks associated with heavy metal exposure. These findings can contribute to informed decision-making, such as implementing appropriate mitigation strategies and establishing guidelines to minimize the risks posed by these contaminants. Further research and monitoring efforts are essential to gain a comprehensive understanding of the extent and implications of heavy metal contamination on human health in these areas. Furthermore, when comparing the results of this study with similar investigations conducted worldwide, it is evident that heavy metal contamination in drinking water is a global concern.

Several studies have reported elevated levels of carcinogenic heavy metals in different regions, emphasizing the need for comprehensive risk assessments and appropriate mitigation strategies^59^. The analysis of cancer risks reveals that certain counties exhibit excess lifetime cancer risks (ELCR) above the acceptable maximum risk suggested by the USEPA, which is ≤ 1 × 10^−6^ (USEPA, 2021). Among the counties studied, Arsanjan, Estheban, and Khoram bid demonstrate higher cancer risks associated with heavy metal exposure. These findings highlight the potential adverse health effects of the investigated heavy metals in drinking water and the importance of implementing measures to reduce their levels. The probable sources of heavy metal contamination in drinking water warrant further investigation.

Although this study did not directly target the identification of specific sources, it is imperative to investigate potential contributors, such as industrial operations, agricultural practices, and geological characteristics. Prior research conducted in diverse regions around the world has pointed to several origins of heavy metal contamination, including mining operations, wastewater discharge, and natural weathering processes. Understanding these sources can aid in implementing targeted interventions to mitigate heavy metal pollution in drinking water. Overall, these mean ELCR values provide valuable information for policymakers, health authorities, and communities to prioritize interventions, regulations, and public health initiatives aimed at minimizing the potential cancer risks associated with heavy metal exposure^4,60,61^.

### Burden of disease

The burden of disease in the assessment of heavy metals (such as As, Pb, Cr, Cd) in drinking water refers to the health impact and negative consequences that arise from the exposure to these specific metals through the consumption of contaminated water. When evaluating the burden of disease, it involves assessing the extent of heavy metal contamination in drinking water sources, estimating the population exposed to these metals, and examining the associated health effects. Heavy metals can have toxic effects on various organ systems in the body, leading to a range of health conditions and diseases. The burden of cancer in the study region was assessed using three key measures: Years of Life Lost (YLL), Years Lived with Disability (YLD), and Disability-Adjusted Life Years (DALY). The results provide important insights into the impact of cancer on premature death and disability in different counties. DALY takes into account not only the years of life lost due to premature death (YLL) but also the years lived with disability (YLD). YLL captures the loss of potential years of life resulting from premature mortality, while YLD quantifies the years lived with a disability or in a less than optimal health state. Table 4 presents the Years of Life Lost (YLL) due to different types of cancer across the counties included in the study^16,20,43^.

**Table 4:**
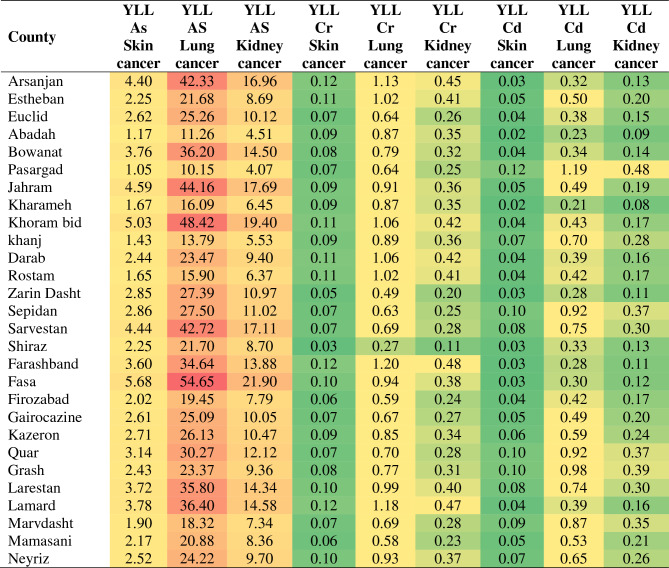
Years of life lost (YLL) due to different types of cancer.

YLL represents the number of years of potential life lost due to premature mortality caused by each type of cancer. It represents the number of years that individuals would have lived if they had not died prematurely due to a specific cause. The table provides specific YLL values for skin cancer, lung cancer, and kidney cancer. The YLL values range from a minimum of 1.05 years for skin cancer to a maximum of 54.65 years for lung cancer. The maximum YLL values indicate that lung cancer has the most significant impact on premature mortality, resulting in a substantial loss of potential years of life. This finding highlights the importance of implementing effective strategies for prevention, early detection, and treatment of lung cancer in the affected counties. The average YLL values provide an overview of the overall impact of cancer on life expectancy in the counties^14,34,42^.

YLD (Years of Life with Disability) measures the burden of non-fatal health outcomes due to a particular condition. It quantifies the number of years lived with a disability caused by a specific disease, such as cancer. Table 5 presents the YLD values for different types of cancer in the studied counties. It provides insight into the specific years lived with disability for each cancer type.

**Table 5:**
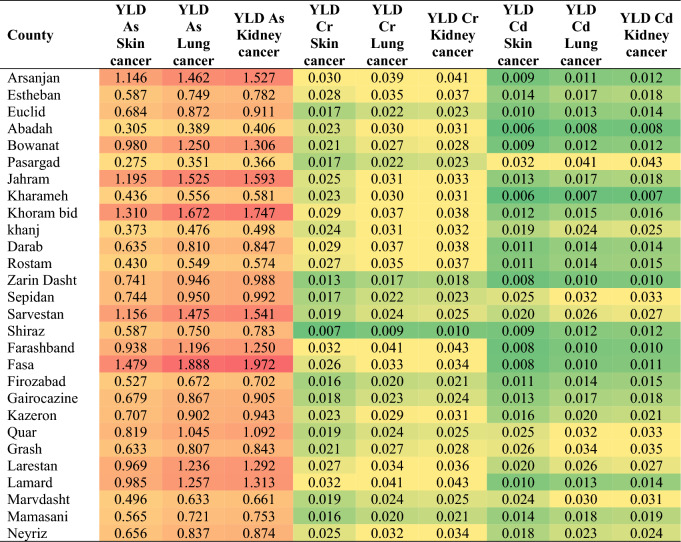
Years of life with disability (YLD) due to different types of cancer.

According to this table, certain regions, such as Arsanjan, Jahram, and Sarvestan, exhibit consistently higher YLD values across all three cancer types. For example, Arsanjan has relatively high YLD values for skin cancer (1.146), lung cancer (1.462), and kidney cancer (1.527). These findings indicate a greater burden of disease and highlight the need for targeted interventions and resources in these areas. When comparing YLD values within each county, lung cancer consistently demonstrates higher YLD values compared to skin and kidney cancer. This suggests that lung cancer may have a more substantial impact on disability within the studied regions. On the other hand, Pasargad and Shiraz have lower YLD values for these cancer types. These differences may indicate variations in disease prevalence, healthcare access, or risk factors among the counties. Understanding these differences can aid in resource allocation and prioritizing healthcare initiatives tailored to the specific needs of each cancer type^47^.

The results of the DALY calculation for three prevalent cancer types across multiple counties have been presemted in Table 6. The table provides a comprehensive view of the disease burden within each region, taking into account both years of life lost due to premature death (YLL) and years lived with disability (YLD).

**Table 6:**
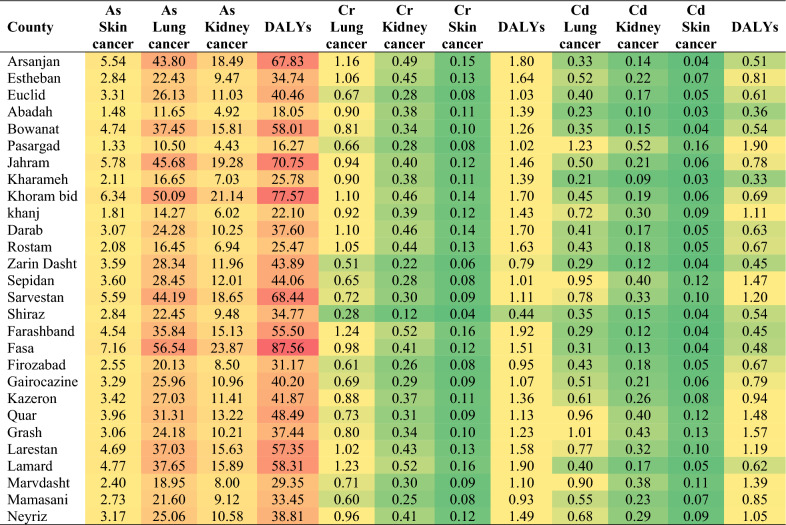
Analysis of disability-adjusted life years (DALY).

As discussed later, the DALY metric combines the YLL and YLD values to provide a measure of the overall burden of disease, reflecting the impact on the population's health in terms of both mortality and disability. Through an examination of the DALY values, we can acquire valuable insights into the comparative burden of skin cancer, lung cancer, and kidney cancer within the counties under study. Based on the Table 6, it is evident that the DALY values vary for different cancer types within each county. The results indicate that among the various counties analyzed, Fasa experiences the greatest burden of disease in terms of DALYs, with a value of 87.56, specifically attributed to cancers caused by exposure to arsenic. DALYs represent the overall impact of a particular health condition, taking into account both years of life lost due to premature mortality and years lived with disability. In this context, Fasa stands out as having a higher number of DALYs compared to other counties, indicating a greater burden of disease associated with arsenic-related cancers. On the other hand, Pasrgad has the lowest DALYs, with a value of 16.27, attributed to cancers caused by exposure to arsenic, indicating a comparatively lower burden of cancer-related disability and premature death. The high value of DALYs in Fasa indicates that despite meeting the recommended limits, heavy metals may still have adverse health effects on the population. The elevated DALYs in Fasa could be attributed to various factors. It is paramount to scrutinize the particular heavy metals found in the drinking water and their respective concentrations. Given that different heavy metals exhibit varying levels of toxicity, even low concentrations can result in cumulative health effects over time. Consequently, an in-depth analysis of the types and concentrations of heavy metals present in Fasa's drinking water is imperative for identifying potential causative agents^1,14,27^.

Furthermore, the health effects of heavy metals can be influenced by various factors, including exposure duration and individual susceptibility. Long-term exposure to low levels of heavy metals can lead to chronic health conditions and contribute to the DALYs observed. Additionally, certain subpopulations, such as children, pregnant women, or individuals with pre-existing health conditions, may be more vulnerable to the adverse effects of heavy metals, resulting in a higher burden of disease. Moreover, considering other potential sources of heavy metal exposure is important. Apart from drinking water, individuals may be exposed to heavy metals through contaminated food, air pollution, or occupational hazards^11,43^.

Assessing these additional exposure pathways can help identify the overall contribution of heavy metals to the DALYs in Fasa and also other studied areas.The table also provides information on DALYs and the corresponding cancers caused by exposure to Cr in drinking water across different counties. The data highlights variations in DALYs and cancer burden among the studied counties. Several observations can be made from the table. First, there is variability in the DALYs across different cancer types. For instance, lung cancer appears to have a higher DALY value compared to kidney and skin cancers. This suggests that Cr exposure in drinking water may have a more substantial impact on lung cancer incidence and associated disability. Farashband stands out with a relatively high DALY value of 1.922, indicating a significant burden of disease attributed to Cr-induced cancers in this area. Other counties, such as Shiraz and Zarin Dasht, exhibit relatively low DALY values. In order to the relatively low DALY values observed in counties like Shiraz and Zarin Dasht, despite exposure to Cr in drinking water, it is possible that the concentration of Cr in the drinking water sources of these counties is comparatively lower than in other areas. Lower Cr levels may result in reduced health risks and, consequently, lower DALY values associated with cancer incidence. Analyzing the specific Cr concentration levels in these counties’ water sources could provide insights into the potential correlation between exposure levels and DALY outcomes^24,56^.

Similarly, about corresponding cancers caused by exposure to Cd in drinking water, the DALY values range from 0.330961 to 1.901714, indicating variations in disease burden. Counties like Zarindasht, Quar, and Grash have higher DALY values, indicating a greater burden of Cd-induced cancers. Factors such as Cd concentration in drinking water, county-specific characteristics, population demographics, and healthcare access contribute to the observed differences in DALY values. Counties with lower DALY values, such as Zarin Dasht, Fasa, and Farashband, may have lower Cd exposure levels or other factors contributing to reduced cancer incidence. Further research, including epidemiological surveys and health risk assessments, is needed to establish causal relationships and identify potential interventions for reducing the burden of Cd-induced cancers in these counties.

In broad terms, it can be asserted that comprehending the burden of disease is a pivotal facet of public health. This understanding necessitates comprehensive assessment and efficient intervention. Through the evaluation of diseases' impact on populations, policymakers and healthcare practitioners can allocate resources judiciously, formulate focused strategies, and institute preventative measures. The burden of disease framework, encompassing both morbidity and mortality, facilitates a comprehensive appraisal of the societal repercussions of diseases. Through continuous research, surveillance, and collaboration, we can strive to alleviate the burden of disease, improve health outcomes, and enhance the overall well-being of individuals and communities^14,44,62^.

## Conclusion

In summary, our study provides a comprehensive assessment of the burden posed by skin cancer, lung cancer, and kidney cancer in the studied counties. By employing the Disability-Adjusted Life Years (DALY) metric, we have gained valuable insights into the overall impact of these cancer types on the population's health, considering both premature deaths and years lived with disability. This analysis revealed notable variations in disease burden across counties, suggesting distinct prevalence rates and impacts of these cancers in different geographic regions. Particularly, Fasa County emerged with a significantly higher burden, highlighting the urgency for targeted interventions and resource allocation to address these pressing health challenges.

Furthermore, our age-specific analysis through DALY calculation offers nuanced insights into how these cancer types affect different age groups within the population. This underscores the importance of tailoring prevention, early detection, and treatment strategies to meet the specific needs of various age cohorts. Effective cancer control measures, including public awareness campaigns, early screening programs, improved healthcare access, and interventions targeting associated risk factors, are crucial in mitigating the burden of skin cancer, lung cancer, and kidney cancer. Overall, this study underscores the critical need for ongoing regional-level surveillance and monitoring of cancer burden. Such insights empower policymakers and healthcare professionals to allocate resources strategically, implement targeted interventions, and formulate effective strategies in the fight against these cancers.

### Supplementary Information

Supplementary Information.

## Data Availability

The data generated and analyzed during this study are available within the study.

## References

[1] Abbasnia A (2019). Prediction of human exposure and health risk assessment to trihalomethanes in indoor swimming pools and risk reduction strategy. Hum. Ecol. Risk Assess. Int. J..

[2] Mirzabeygi M (2017). Heavy metal contamination and health risk assessment in drinking water of Sistan and Baluchistan, Southeastern Iran. Hum. Ecol. Risk Assess. Int. J..

[3] Noh J (2020). Estimating the disease burden of lung cancer attributable to residential radon exposure in Korea during 2006–2015: A socio-economic approach. Sci Total Environ.

[4] Radfard M (2019). Drinking water quality and arsenic health risk assessment in Sistan and Baluchestan, Southeastern Province, Iran. Hum. Ecol. Risk Assess. Int. J..

[5] Sener E, Sener S, Bulut C (2023). Assessment of heavy metal pollution and quality in lake water and sediment by various index methods and GIS: A case study in Beysehir Lake, Turkey. Mar Pollut Bull.

[6] Cotruvo JA (2017). 2017 WHO guidelines for drinking water quality: First addendum to the fourth edition. J. Am. Water Works Assoc..

[7] Mawari G (2022). Human Health risk assessment due to heavy metals in ground and surface water and association of diseases with drinking water sources: A study from Maharashtra, India. Environ. Health Insights.

[8] World Health Organization (2002). Guidelines for Drinking-Water Quality.

[9] Sharafi K (2022). Investigation of health risk assessment and the effect of various irrigation water on the accumulation of toxic metals in the most widely consumed vegetables in Iran. Sci. Rep..

[10] Soleimani H (2022). Groundwater quality evaluation and risk assessment of nitrate using monte carlo simulation and sensitivity analysis in rural areas of Divandarreh County, Kurdistan province, Iran. Int. J. Environ. Anal. Chem..

[11] Collaborators GBDB (2018). Burden of disease in Brazil, 1990–2016: A systematic subnational analysis for the Global Burden of Disease Study 2016. Lancet.

[12] Soleimani H (2022). Probabilistic and deterministic approaches to estimation of non-carcinogenic human health risk due to heavy metals in groundwater resources of torbat heydariyeh, southeastern of Iran. Int. J. Environ. Anal. Chem..

[13] GBD 2017 Risk Factor Collaborators (2018). Global, regional, and national comparative risk assessment of 84 behavioural, environmental and occupational, and metabolic risks or clusters of risks for 195 countries and territories, 1990–2017: A systematic analysis for the Global Burden of Disease Study 2017. Lancet.

[14] Naddafi K (2022). Assessment of burden of disease induced by exposure to heavy metals through drinking water at national and subnational levels in Iran, 2019. Environ. Res..

[15] Zhai C (2023). Global, regional, and national deaths, disability-adjusted life years, years lived with disability, and years of life lost for the global disease burden attributable to second-hand smoke, 1990–2019: A systematic analysis for the Global Burden of Disease Study. Sci. Total Environ..

[16] Abtahi M (2019). Age-sex specific disability-adjusted life years (DALYs) attributable to elevated levels of fluoride in drinking water: A national and subnational study in Iran, 2017. Water Res..

[17] Badeenezhad A (2020). Estimation of the groundwater quality index and investigation of the affecting factors their changes in Shiraz drinking groundwater, Iran. Groundw. Sustain. Dev..

[18] Zhang Y (2023). Global burden of tracheal, bronchus, and lung cancer attributable to occupational carcinogens in 204 countries and territories, from 1990 to 2019: Results from the global burden of disease study 2019. Ann. Med..

[19] Abtahi M (2023). Assessment of cause-specific mortality and disability-adjusted life years (DALYs) induced by exposure to inorganic arsenic through drinking water and foodstuffs in Iran. Sci. Total Environ..

[20] GBD 2015 Risk Factor Collaborators Group (2016). Global, regional, and national comparative risk assessment of 79 behavioural, environmental and occupational, and metabolic risks or clusters of risks, 1990–2015: A systematic analysis for the Global Burden of Disease Study 2015. Lancet.

[21] Filippelli G (2020). New approaches to identifying and reducing the global burden of disease from pollution. Geohealth.

[22] Rice EW, Bridgewater L, Association APH (2012). Standard Methods for the Examination of Water and Wastewater.

[23] Azhdarpoor A (2019). Assessing fluoride and nitrate contaminants in drinking water resources and their health risk assessment in a semiarid region of southwest Iran. Desalin. Water Treat..

[24] Collaborators GBDI (2022). Health system performance in Iran: A systematic analysis for the Global Burden of Disease Study 2019. Lancet.

[25] Marufi, N., *et al.* Carcinogenic and non-carcinogenic human health risk assessments of heavy metals contamination in drinking water supplies in Iran: A systematic review. *Rev. Environ. Health* (2022). 10.1515/reveh-2022-0060 36181734

[26] Moya, J., *et al.**Exposure Factors Handbook, 2011 Edition*. US Environmental Protection Agency, Washington. (2011), EPA/600/R-090.

[27] GBD 2019 Risk Factor Collaborators (2022). The global burden of cancer attributable to risk factors, 2010–19: A systematic analysis for the Global Burden of Disease Study 2019. Lancet.

[28] Peana M (2022). Biological effects of human exposure to environmental cadmium. Biomolecules.

[29] Badeenezhad A (2021). Effect of land use changes on non-carcinogenic health risks due to nitrate exposure to drinking groundwater. Environ. Sci. Pollut. Res..

[30] Badeenezhad A (2021). Factors affecting the nitrate concentration and its health risk assessment in drinking groundwater by application of Monte Carlo simulation and geographic information system. Hum. Ecol. Risk Assess. Int. J..

[31] Jamshidi A (2021). Water quality evaluation and non-cariogenic risk assessment of exposure to nitrate in groundwater resources of Kamyaran, Iran: Spatial distribution, Monte-Carlo simulation, and sensitivity analysis. J. Environ. Health Sci. Eng..

[32] Phillips L, Moya J (2013). The evolution of EPA’s Exposure Factors Handbook and its future as an exposure assessment resource. J. Expo. Sci. Environ. Epidemiol..

[33] USEPA (2007). Guidance for evaluating the oral bioavailability of metals in soils for use in human health risk assessment. OSWER.

[34] Monteiro De Oliveira EC (2021). Arsenic exposure from groundwater: Environmental contamination, human health effects, and sustainable solutions. J. Toxicol. Environ. Health B Crit. Rev..

[35] Mohammadi AA (2018). Using the combined model of gamma test and neuro-fuzzy system for modeling and estimating lead bonds in reservoir sediments. Environ. Sci. Pollut. Res..

[36] US Environmental Protection Agency. *Guidance for Performing Aggregate Exposure and Risk Assessments*. (US Environmental Protection Agency, Office of Pesticide Programs Washington (DC), 1999).

[37] US Environmental Protection Agency (2007). Guidance for evaluating the oral bioavailability of metals in soils for use in human health risk assessment. OSWER.

[38] VEAL, L., *United States Environmental Protection Agency*. 2021.

[39] Steel N (2018). Changes in health in the countries of the UK and 150 English Local Authority areas 1990–2016: A systematic analysis for the Global Burden of Disease Study 2016. Lancet.

[40] Wu P (2023). Systematic analysis and prediction for disease burden of ovarian cancer attributable to hyperglycemia: A comparative study between China and the world from 1990 to 2019. Front. Med. (Lausanne).

[41] KazemiMoghaddam V (2022). Heavy metal contaminated soil, water, and vegetables in northeastern Iran: Potential health risk factors. J. Environ. Health Sci. Eng..

[42] Diseases GBD, Injuries C (2020). Global burden of 369 diseases and injuries in 204 countries and territories, 1990–2019: A systematic analysis for the Global Burden of Disease Study 2019. Lancet.

[43] Dobaradaran S (2020). Age-sex specific and cause-specific health risk and burden of disease induced by exposure to trihalomethanes (THMs) and haloacetic acids (HAAs) from drinking water: An assessment in four urban communities of Bushehr Province, Iran, 2017. Environ. Res..

[44] Liu H (2023). Global disease burden and attributable risk factor analysis of asthma in 204 countries and territories from 1990 to 2019. Allergy Asthma Immunol. Res..

[45] Jafarzadeh N (2022). Non-carcinogenic risk assessment of exposure to heavy metals in underground water resources in Saraven, Iran: Spatial distribution, monte-carlo simulation, sensitive analysis. Environ. Res..

[46] Wolf J (2023). Burden of disease attributable to unsafe drinking water, sanitation, and hygiene in domestic settings: A global analysis for selected adverse health outcomes. Lancet.

[47] Liu J (2022). The burden of coronary heart disease and stroke attributable to dietary cadmium exposure in Chinese adults, 2017. Sci. Total Environ..

[48] Xu Q (2023). Epidemiological trends of kidney cancer along with attributable risk factors in China from 1990 to 2019 and its projections until 2030: An analysis of the global burden of disease study 2019. Clin. Epidemiol..

[49] Wu S (2023). Global burden of cardiovascular disease attributable to metabolic risk factors, 1990–2019: An analysis of observational data from a 2019 Global Burden of Disease study. BMJ Open.

[50] Kiani B (2021). Association between heavy metals and colon cancer: an ecological study based on geographical information systems in North-Eastern Iran. BMC Cancer.

[51] Liu W (2023). Global, regional, and national burden of chronic kidney disease attributable to high sodium intake from 1990 to 2019. Front. Nutr..

[52] Redondo HG (2023). Harmonized approach to estimate the burden of disease of dietary exposure to four chemical contaminants—A French study. Sci. Total Environ..

[53] Golaki M (2022). Health risk assessment and spatial distribution of nitrate, nitrite, fluoride, and coliform contaminants in drinking water resources of Kazerun, Iran. Environ. Res..

[54] Habib, S. S., et al., Assessment and bioaccumulation of heavy metals in water, fish (wild and farmed) and associated human health risk. *Biol. Trace Elem. Res.* (2023). 10.1007/s12011-023-03703-2 37178449

[55] Hasan AB (2023). Spatial distribution, water quality, human health risk assessment, and origin of heavy metals in groundwater and seawater around the ship-breaking area of Bangladesh. Environ. Sci. Pollut. Res. Int..

[56] Kilavi PK (2021). Quality and human health risk assessment of uranium and other heavy metals in drinking water from Kwale County, Kenya. Environ. Monit. Assess..

[57] Zeng X (2023). Heavy metal risk of disposable food containers on human health. Ecotoxicol. Environ. Saf..

[58] Gupta S, Gupta SK (2023). Application of Monte Carlo simulation for carcinogenic and non-carcinogenic risks assessment through multi-exposure pathways of heavy metals of river water and sediment, India. Environ. Geochem. Health.

[59] Banerjee S (2023). Investigating the synergistic role of heavy metals in Arsenic-induced skin lesions in West Bengal, India. J. Trace Elem. Med. Biol..

[60] Khan R (2021). Environmental contamination by heavy metals and associated human health risk assessment: A case study of surface water in Gomti River Basin, India. Environ. Sci. Pollut. Res. Int..

[61] Khan S (2013). Drinking water quality and human health risk in Charsadda district, Pakistan. J. Clean. Prod..

[62] Emmanuel UC (2022). Human health risk assessment of heavy metals in drinking water sources in three senatorial districts of Anambra State, Nigeria. Toxicol. Rep..

